# Synostosis of the interphalangeal joint: an uncommon cause of post-fracture digital stiffness

**DOI:** 10.1080/23320885.2021.1935966

**Published:** 2021-06-14

**Authors:** Peter Y. W. Chan, Peter S. H. Chan

**Affiliations:** Hand Surgery Specialists, LLC, Morristown, NJ, USA

**Keywords:** Synostosis, heterotopic ossification, post-fracture, stiffness, interphalangeal joint

## Abstract

Loss of motion and stiffness after fracture of the digits are most commonly a result of soft tissue contracture and adhesions. However, stiffness can also have a bony etiology. We present a case of synostosis of the thumb interphalangeal joint after non-operative treatment of a closed fracture.

## Introduction

Stiffness and loss of motion are common sequelae after fractures of the phalanges of the hand. Fibrosis and contracture of collateral ligaments, joint capsule, and/or adhesions of the flexor and extensor tendons are common causes of stiffness and can result in significant loss of function. Since a soft tissue etiology is most common, bony causes of post-traumatic stiffness may be overlooked. Although uncommon in the hand, synostosis may be a late cause of stiffness after injury. We present a case of synostosis of the thumb after closed fracture.

## Case report

**Figure 1.**
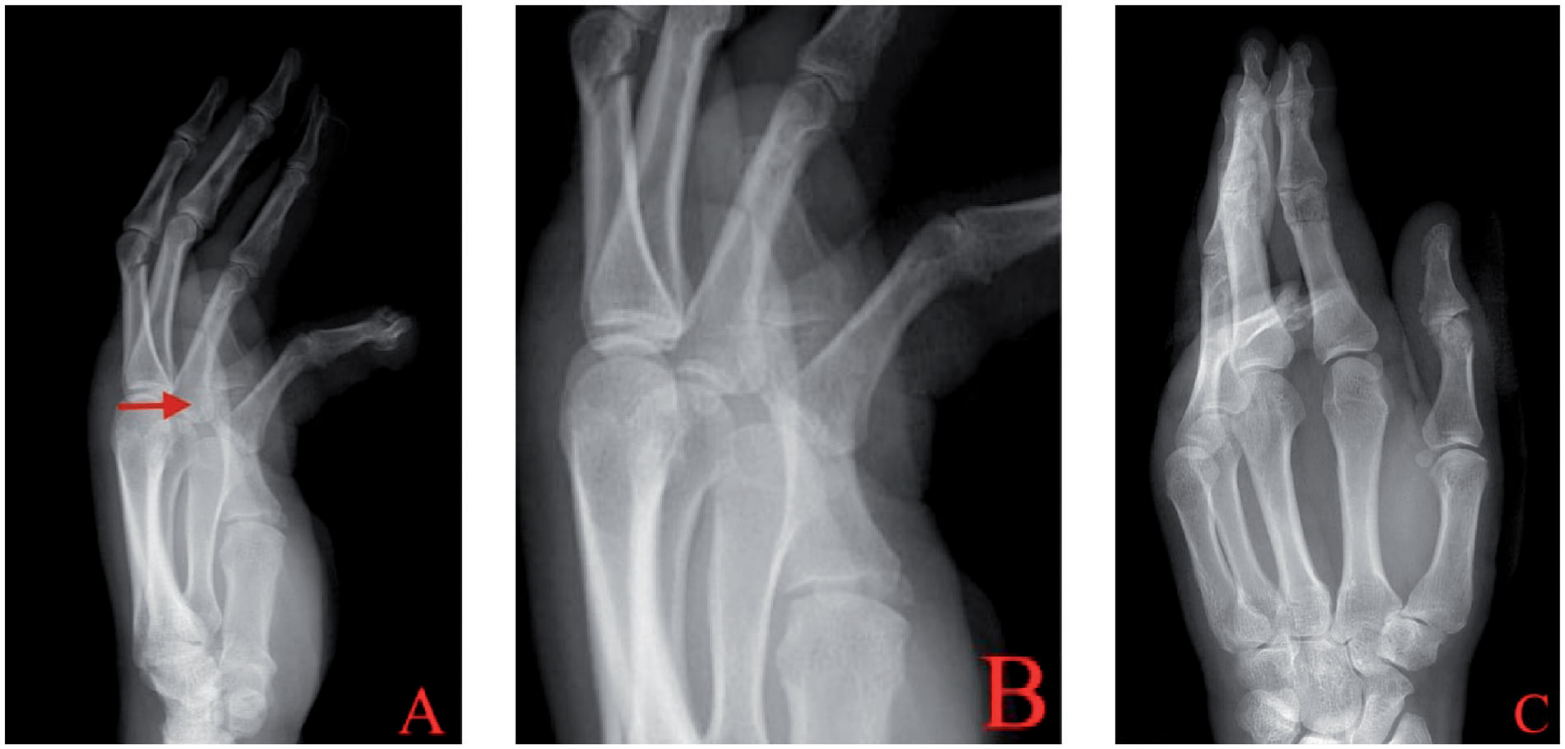
Injury radiographs anteroposterior and lateral of hand (A, C) with focus on thumb (B) demonstrate a condylar fracture of proximal phalangeal head (arrow).

The patient is a 48-year-old right-hand dominant male who injured his left hand reaching into a snow blower. The patient was taken in transfer from an outside hospital arriving with plain films from the facility’s emergency department. Physical exam and the plain films revealed closed injuries including a minimally displaced radial condylar fracture of the thumb proximal phalangeal head, minimally displaced supracondylar neck fracture of the index finger proximal phalanx, nondisplaced midshaft fracture of the middle phalanx of the middle finger and an open fracture dislocation through the distal interphalangeal (DIP) joint of the small finger with near complete amputation (Figure 1).

There was a delay in transfer resulting in the patient presenting at almost 6 h post-injury. The patient was anxious to proceed with treatment. The patient’s injuries were identifiable from the plain films and due to the open nature of the small finger injury, repeat films were not obtained before surgery. The emergent issue was the open injury to the small finger and treatment options were discussed with the patient. Revision amputation was chosen as treatment for the small finger. Closed treatment without reduction was chosen for the index and middle fingers secondary to the stable nature of the fractures. The intra-articular nature of the thumb fracture was discussed with the patient, including the risk of post-traumatic arthritis. However, the patient declined surgical treatment. Therefore, closed treatment without reduction was chosen as definitive management.

Revision amputation with disarticulation through the DIP joint and primary closure of the left small finger injury was performed. The patient began a course of hand therapy at 5 days post-injury. A hand-based thumb spica splint was fabricated including the interphalangeal (IP) joint of the thumb in neutral extension at metacarpophalangeal (MP) and IP joint. The index finger MP and proximal interphalangeal (PIP) joint were included in the splint in intrinsic plus position and the middle finger included the DIP joint, also in intrinsic plus position. Secondary to the stable nature of the index and middle finger fractures, we started active and active assistive motion to those digits. We allowed for unrestricted active and gentle passive motion to the ring and small fingers. As the thumb proximal phalangeal head fracture was intra-articular, it was decided to delay range of motion for 3 weeks until there was clinical union with no pain to palpation at the fracture site.

**Figure 2.**
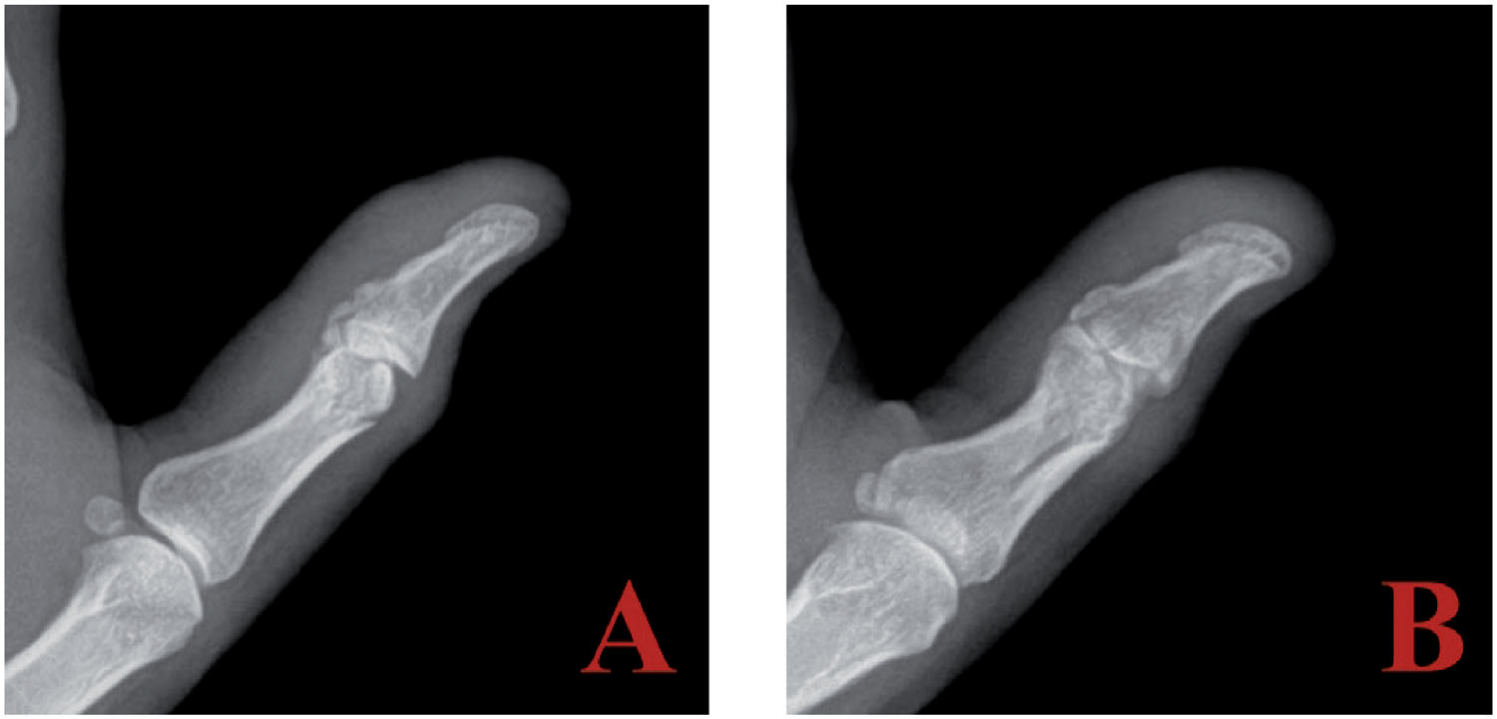
Plain radiograph lateral and oblique (A, B) of thumb two months post injury indicate early HO developing at the IP joint.

**Figure 3.**
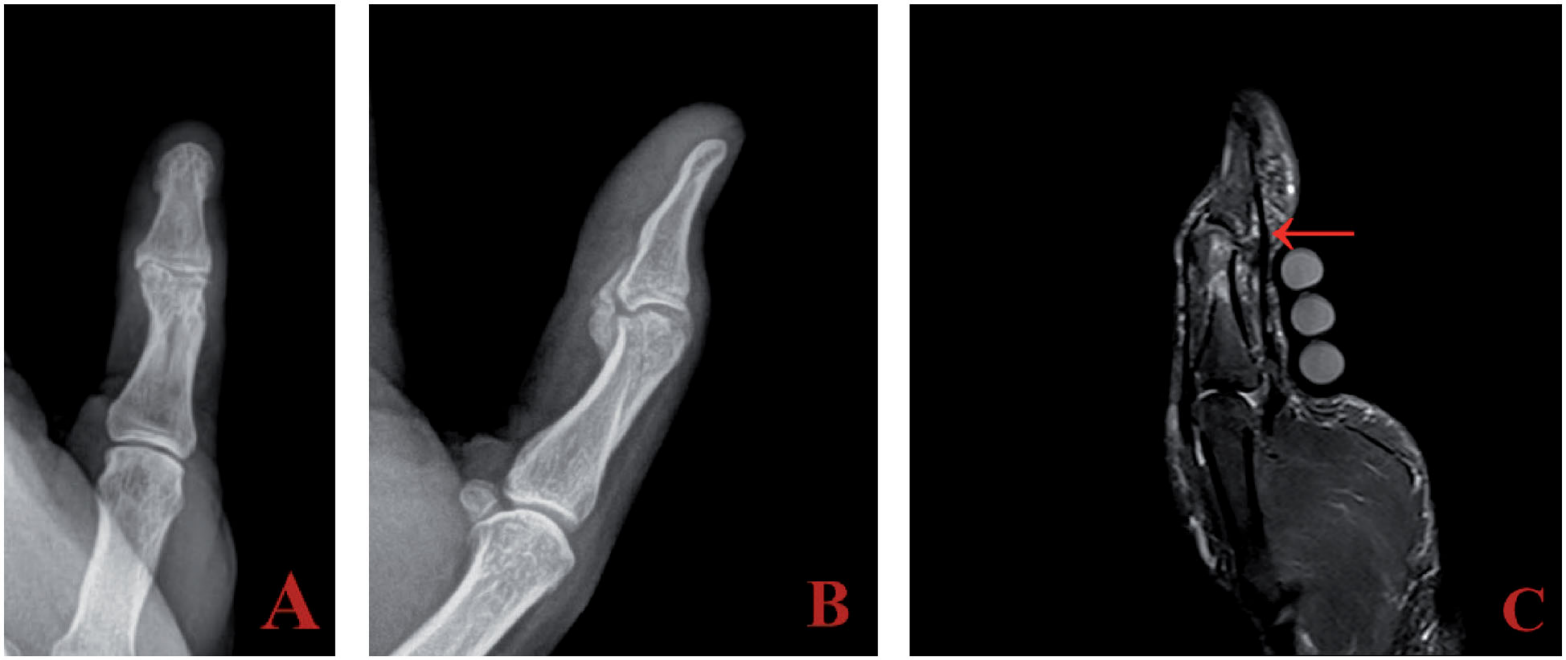
Anteroposterior (A) and lateral (B) views at eight months post injury show HO progression into synostosis. Sagittal STIR image on MRI (C) demonstrates an intact flexor tendon (red arrow) without involvement in synostosis.

Upon discontinuation of immobilization of the thumb at three weeks post-injury, the patient’s arc of motion at the IP joint actively and passively was 25°. Within the 3 and 8-week post-injury time period, the therapist reported worsening of active and passive motion. At 8-week follow up, the patient lacked active and passive motion at the IP joint; the joint was fixed at 20° of flexion. Range of motion had normalized in all other fingers. All fractures were healed radiographically; however, early heterotopic ossification (HO) was noted at the IP joint of the thumb (Figure 2). The calcification was in the anatomic location of the volar plate and had a lamellar appearance. Progression of the HO to mature synostosis involving the volar plate and a portion of the accessory collateral ligaments was identified radiographically eight months post-injury (Figure 3). Specifically, the HO was seen spanning the IP joint, fusing the proximal and distal phalanges.

To rule out flexor tendon discontinuity as a cause of loss of motion, an MRI scan was obtained which revealed an intact flexor tendon with no bony involvement of the tendon (Figure 3). The loss of motion was not associated with pain or tenderness to palpation. Secondary to the complete loss of motion and radiographic maturity of the synostosis, surgical treatment was offered to the patient.

**Figure 4.**
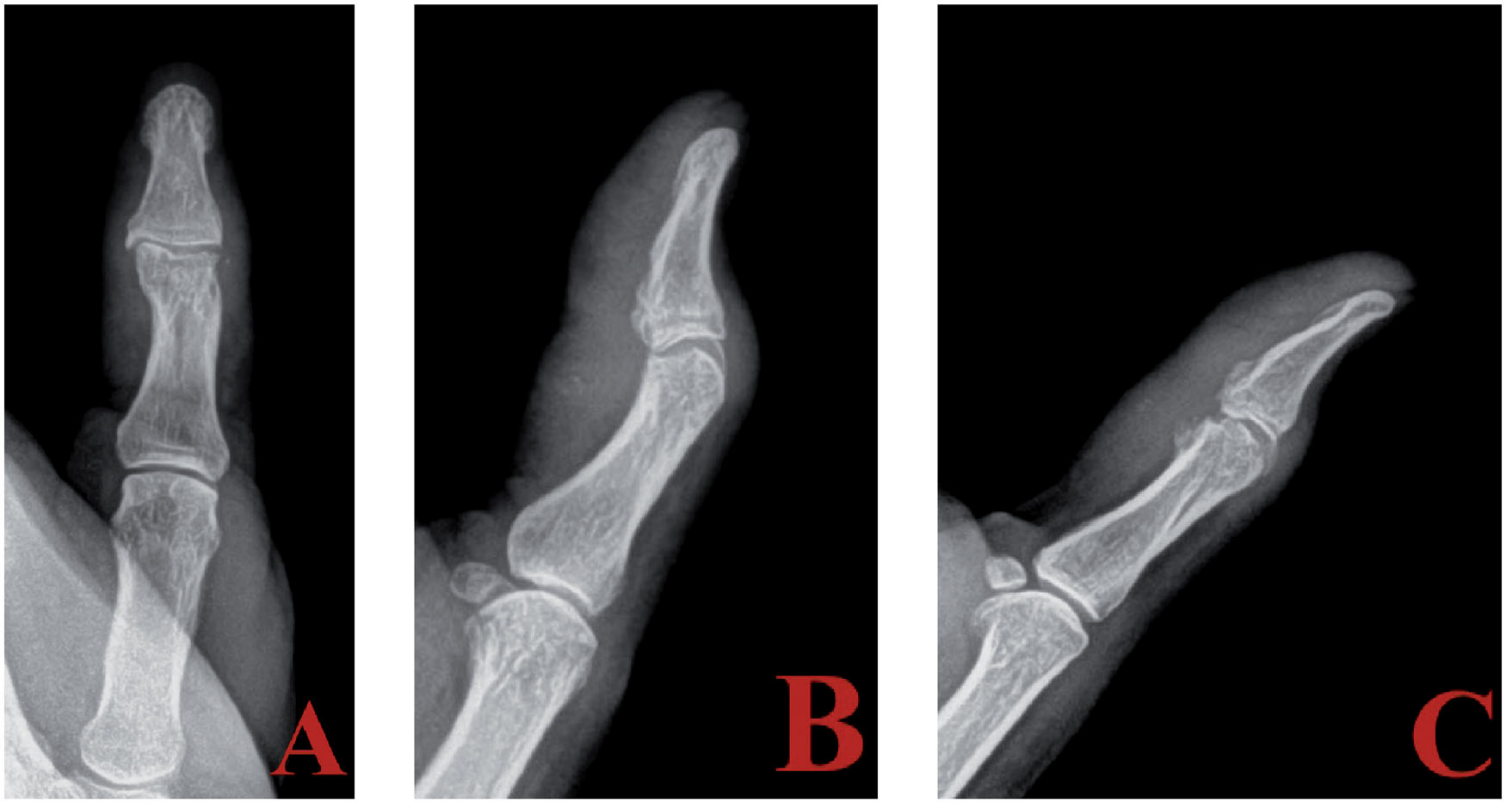
Anteroposterior (A), lateral (B), and oblique (C) views post excision of synostosis.

Excision of the synostosis was performed through a volar approach (Figure 4). Mobilization of the digital nerves and arteries was achieved. The distal portion of the A2 pulley was incised allowing for retraction of the flexor tendon. The synostosis involved the volar plate as well as the accessory collateral ligaments, which were excised with a combination of osteotomes and rongeur. The proper collateral ligaments were not involved and therefore were not incised. There was no instability to either radial ulnar or dorsal volar stress after bony excision. No ligament reconstruction was needed. There was no evidence of impingement or intra-articular adhesions after excision of the bony mass; passive motion was essentially full. The patient was placed on indomethacin postoperatively for 6 weeks. He resumed hand therapy at 2 days post-surgery with no use of a splint and an active and passive motion protocol with unrestricted strengthening. At 8 weeks, he had regained a functional arc of motion of 60° at the IP joint. At last follow up 6 months post-resection, he had no evidence of recurrence.

## Discussion

Fractures of the tubular bones of the hand are common injuries with an incidence of 12.5 per 10,000 persons per year for the phalanges [1]. The primary goal of treating hand fractures is regaining early range of motion, facilitating a quicker return to function. Treated either non-operatively with functional bracing or with open reduction internal fixation, early mobilization is imperative to avoid stiffness [2].

Post-traumatic stiffness of the hand is typically a result of fibrosis and contracture of soft tissue structures including the collateral ligaments, volar plate, and tendon adhesions. Stiffness of the hand can occur as a result of heterotopic ossification and synostosis, but is rare. Heterotopic ossification (HO) is characterized by the presence of bone in soft tissue where bone normally does not exist. Synostosis represents the progression of HO resulting in the fusion of two bones. In our case, the initial calcification represented HO which progressed into synostosis once the IP joint was fused.

HO and synostosis are often associated with closed head, spinal cord, and thermal injuries [3–6]. In the appendicular skeletal system, large joints are more commonly affected. Development of HO in the hand is rare but has been described. Henderson et al. described two cases of HO formation, one following head injury and the other following spinal cord injury [4]. The former presented with HO formation in the tendon sheaths and tendons of the index, middle, and ring finger in addition to other large joints including the hip and elbow. The latter presented with HO in the left middle and ring finger as well as the right middle finger. Meythaler et al. reported a case of HO in the extensor tendons of the index, middle, and ring fingers after spinal cord injury. HO developed in the extensor sheaths of the digits, ranging from the metacarpal-phalangeal joint to the proximal interphalangeal joint [5]. Synostosis, defined as bony fusion of a joint, in the digits is significantly more rare. Asselmeir et al. described a case of synostosis of the ring and middle finger proximal interphalangeal joints after a closed head injury without fracture of the digits [6].

HO has been described after open injuries to the hand, specifically lacerations. Barlaan et al. described a case of HO after traumatic laceration of the middle finger extensor tendon without bony injury [7]. Lorio et al. described the development of HO after flexor tendon laceration of the left index finger also without bony injury [8].

HO in the hand has been reported without a history of trauma. Hamada et al. described the development of HO in the digital nerves [9]. Kim et al. reported the development of HO as a result of ossifying fasciitis in the flexor tendon sheaths of the palm [10]. As far as the authors are aware, no literature has described synostosis in the thumb. Our case is unique in that it describes the progression of HO into synostosis in the thumb after closed fracture but in the absence of neural or thermal injury or open injury to the digit.

Orthopedists should be cognizant that persistent late stiffness may be a result of a bony etiology and not just soft tissue fibrosis and contracture. Impingement by heterotopic bone may result in some loss of motion; however, complete loss of passive motion may suggest synostosis. Intra-articular malunion or involvement of the retrocondylar space with mechanical impingement can also result in loss of active and passive motion. While there was joint incongruity in our example, the patient regained functional range of motion after removal of the bony bridge, indicating the stiffness was the result of the synostosis and not articular malunion or adhesions.

Other etiology should be considered when confronted with radiographic heterotopic bone or periarticular calcification. Acute calcific periarthritis, gout and pseudo-gout can present with calcification on imaging studies, which is typically amorphous. These inflammatory arthropathies typically present as atraumatic and painful. Radiographic findings typically will resolve or improve in 2–3 weeks [11]. Our patient did not present with pain outside of the initial traumatic injury and the bony structure displayed lamellar calcification that matured and persisted.

Experience with early excision of synostosis at the proximal radioulnar joint of the elbow suggests that waiting for radiographic maturity is not needed [12]. Early excision between 6 and 12 months without radiographic maturity did not increase risk of recurrence. The authors state that associated soft tissue contracture may be minimized with early resection. In our case, we felt that waiting for radiographic maturity was prudent as any associated soft tissue structures, specifically collateral ligaments and dorsal capsule, could be released simply at the time of resection.

Indomethacin was prescribed postoperatively for prophylaxis against recurrence. While multiple studies have compared the efficacy of the use of indomethacin and radiation therapy for the prevention of HO, these studies predominantly focus on the hip [13–17]. Multiple studies have concluded that indomethacin and radiation are equally effective in the prevention of HO development after acetabular fractures and hip arthroplasty. Contraindications of indomethacin use are a history of ulcers and renal dysfunction while radiation treatment for HO prophylaxis has demonstrated the potential for development of osteosarcoma [18, 19]. To this point, no studies have compared indomethacin and radiation use within the arm or hand. Our patient was young with no contraindications for nonsteroidal anti-inflammatory drug use. Therefore, because of the comparable effectiveness of the two therapies within other body sites, indomethacin was chosen over radiation therapy.
